# Supplementary material for: Cancer incidence estimation from mortality data: a validation study within a population-based cancer registry
Source: Popul Health Metr. 2021 Mar 23;19:18. doi: 10.1186/s12963-021-00248-1 (PMC7988947; doi:10.1186/s12963-021-00248-1)
Supplement: Supplementary file 1 — Additional file 1: Table S1. Observed cases and expected cases under each scenario, and goodness of fit measures. Men. [file 12963_2021_248_MOESM1_ESM.docx]

**Table S1.** Observed cases and expected cases under each scenario, and goodness of fit measures. Men.

| Cancer site | Scenario | 2004 | 2005 | 2006 | 2007 | 2008 | 2009 | 2010 | 2011 | 2012 | 2013 | MAPE |
| --- | --- | --- | --- | --- | --- | --- | --- | --- | --- | --- | --- | --- |
| Colon | O | 138 | 203 | 156 | 180 | 172 | 227 | 208 | 214 | 243 | 222 |  |
|  | E(C1) | 138 | 172 | 179 | 164 | 195 | 186 | 222 | 211 | 215 | 230 |  |
|  |  | (0.0%) | (15.3%) | (14.7%) | (8.9%) | (13.4%) | (18.1%) | (6.7%) | (1.4%) | (11.5%) | (3.6%) | 9.36 |
|  | E(C3) | 136 | 165 | 173 | 163 | 190 | 189 | 215 | 211 | 217 | 229 |  |
|  |  | (1.4%) | (18.7%) | (10.9%) | (9.4%) | (10.5%) | (16.7%) | (3.4%) | (1.4%) | (10.7%) | (3.2%) | 8.63 |
|  | E(C5) | 133 | 158 | 167 | 149 | 180 | 174 | 206 | 194 | 203 | 214 |  |
|  |  | (3.6%) | (22.2%) | (7.1%) | (17.2%) | (4.7%) | (23.3%) | (1.0%) | (9.3%) | (16.5%) | (3.6%) | 10.84 |
|  | E(L) | 141 | 182 | 187 | 178 | 207 | 199 | 233 | 227 | 228 | 245 |  |
|  |  | (2.2%) | (10.3%) | (19.9%) | (1.1%) | (20.3%) | (12.3%) | (12.0%) | (6.1%) | (6.2%) | (10.4%) | 10.08 |
|  | E(Q) | 151 | 230 | 232 | 161 | 231 | 158 | 281 | 207 | 193 | 229 |  |
|  |  | (9.4%) | (13.3%) | (48.7%) | (10.6%) | (34.3%) | (30.4%) | (35.1%) | (3.3%) | (20.6%) | (3.2%) | 20.88 |
| Rectum | O | 90 | 97 | 93 | 95 | 115 | 136 | 112 | 110 | 115 | 121 |  |
|  | E(C1) | 68 | 74 | 71 | 90 | 127 | 105 | 137 | 135 | 96 | 112 |  |
|  |  | (24.4%) | (23.7%) | (23.7%) | (5.3%) | (10.4%) | (22.8%) | (22.3%) | (22.7%) | (16.5%) | (7.4%) | 17.93 |
|  | E(C3) | 71 | 73 | 69 | 87 | 118 | 101 | 131 | 129 | 96 | 110 |  |
|  |  | (21.1%) | (24.7%) | (25.8%) | (8.4%) | (2.6%) | (25.7%) | (17.0%) | (17.3%) | (16.5%) | (9.1%) | 16.83 |
|  | E(C5) | 62 | 65 | 66 | 79 | 107 | 98 | 120 | 119 | 93 | 92 |  |
|  |  | (31.1%) | (33.0%) | (29.0%) | (16.8%) | (7.0%) | (27.9%) | (7.1%) | (8.2%) | (19.1%) | (24.0%) | 20.33 |
|  | E(L) | 76 | 83 | 75 | 100 | 143 | 109 | 151 | 147 | 99 | 132 |  |
|  |  | (15.6%) | (14.4%) | (19.4%) | (5.3%) | (24.3%) | (19.9%) | (34.8%) | (33.6%) | (13.9%) | (9.1%) | 19.03 |
|  | E(Q) | 47 | 73 | 89 | 107 | 216 | 147 | 182 | 176 | 95 | 111 |  |
|  |  | (47.8%) | (24.7%) | (4.3%) | (12.6%) | (87.8%) | (8.1%) | (62.5%) | (60.0%) | (17.4%) | (8.3%) | 33.35 |
| Lung | O | 307 | 350 | 332 | 344 | 377 | 317 | 348 | 312 | 353 | 351 |  |
|  | E(C1) | 307 | 346 | 338 | 326 | 348 | 324 | 320 | 366 | 362 | 353 |  |
|  |  | (0.0%) | (1.1%) | (1.8%) | (5.2%) | (7.7%) | (2.2%) | (8.0%) | (17.3%) | (2.5%) | (0.6%) | 4.66 |
|  | E(C3) | 310 | 343 | 331 | 322 | 341 | 319 | 313 | 356 | 354 | 350 |  |
|  |  | (1.0%) | (2.0%) | (0.3%) | (6.4%) | (9.5%) | (0.6%) | (10.1%) | (14.1%) | (0.3%) | (0.3%) | 4.46 |
|  | E(C5) | 313 | 345 | 336 | 323 | 333 | 313 | 299 | 346 | 343 | 335 |  |
|  |  | (2.0%) | (1.4%) | (1.2%) | (6.1%) | (11.7%) | (1.3%) | (14.1%) | (10.9%) | (2.8%) | (4.6%) | 5.60 |
|  | E(L) | 303 | 344 | 336 | 327 | 358 | 331 | 336 | 379 | 375 | 369 |  |
|  |  | (1.3%) | (1.7%) | (1.2%) | (4.9%) | (5.0%) | (4.4%) | (3.4%) | (21.5%) | (6.2%) | (5.1%) | 5.49 |
|  | E(Q) | 281 | 365 | 394 | 357 | 399 | 359 | 363 | 442 | 417 | 372 |  |
|  |  | (8.5%) | (4.3%) | (18.7%) | (3.8%) | (5.8%) | (13.2%) | (4.3%) | (41.7%) | (18.1%) | (6.0%) | 12.44 |
| Prostate | O | 327 | 351 | 405 | 442 | 478 | 498 | 463 | 519 | 494 | 447 |  |
|  | E(C1) | 224 | 295 | 269 | 242 | 266 | 340 | 330 | 325 | 486 | 663 |  |
|  |  | (31.5%) | (16.0%) | (33.6%) | (45.2%) | (44.4%) | (31.7%) | (28.7%) | (37.4%) | (1.6%) | (48.3%) | 31.84 |
|  | E(C3) | 216 | 271 | 251 | 221 | 247 | 312 | 309 | 301 | 436 | 558 |  |
|  |  | (33.9%) | (22.8%) | (38.0%) | (50.0%) | (48.3%) | (37.3%) | (33.3%) | (42.0%) | (11.7%) | (24.8%) | 34.23 |
|  | E(C5) | 160 | 192 | 181 | 159 | 173 | 218 | 218 | 214 | 306 | 356 |  |
|  |  | (51.1%) | (45.3%) | (55.3%) | (64.0%) | (63.8%) | (56.2%) | (52.9%) | (58.8%) | (38.1%) | (20.4%) | 50.58 |
|  | E(L) | 296 | 414 | 370 | 334 | 374 | 484 | 462 | 455 | 694 | 1,053 |  |
|  |  | (9.5%) | (17.9%) | (8.6%) | (24.4%) | (21.8%) | (2.8%) | (0.2%) | (12.3%) | (40.5%) | (135.6%) | 27.37 |
|  | E(Q) | 250 | 468 | 388 | 410 | 383 | 547 | 468 | 496 | 932 | 1,948 |  |
|  |  | (23.5%) | (33.3%) | (4.2%) | (7.2%) | (19.9%) | (9.8%) | (1.1%) | (4.4%) | (88.7%) | (335.8%) | 52.80 |
| Bladder | O | 257 | 245 | 245 | 253 | 251 | 275 | 264 | 265 | 312 | 268 |  |
|  | E(C1) | 234 | 226 | 266 | 305 | 259 | 259 | 275 | 243 | 268 | 259 |  |
|  |  | (8.9%) | (7.8%) | (8.6%) | (20.6%) | (3.2%) | (5.8%) | (4.2%) | (8.3%) | (14.1%) | (3.4%) | 8.48 |
|  | E(C3) | 230 | 223 | 267 | 301 | 270 | 263 | 278 | 242 | 263 | 254 |  |
|  |  | (10.5%) | (9.0%) | (9.0%) | (19.0%) | (7.6%) | (4.4%) | (5.3%) | (8.7%) | (15.7%) | (5.2%) | 9.43 |
|  | E(C5) | 205 | 202 | 239 | 273 | 249 | 248 | 271 | 239 | 253 | 237 |  |
|  |  | (20.2%) | (17.6%) | (2.4%) | (7.9%) | (0.8%) | (9.8%) | (2.7%) | (9.8%) | (18.9%) | (11.6%) | 10.17 |
|  | E(L) | 262 | 247 | 294 | 334 | 275 | 271 | 281 | 247 | 279 | 277 |  |
|  |  | (1.9%) | (0.8%) | (20.0%) | (32.0%) | (9.6%) | (1.5%) | (6.4%) | (6.8%) | (10.6%) | (3.4%) | 9.30 |
|  | E(Q) | 252 | 239 | 246 | 327 | 182 | 221 | 251 | 253 | 303 | 286 |  |
|  |  | (1.9%) | (2.4%) | (0.4%) | (29.2%) | (27.5%) | (19.6%) | (4.9%) | (4.5%) | (2.9%) | (6.7%) | 10.02 |
| Stomach | O | 87 | 70 | 89 | 76 | 94 | 76 | 83 | 67 | 86 | 73 |  |
|  | E(C1) | 80 | 64 | 72 | 89 | 73 | 74 | 89 | 82 | 76 | 103 |  |
|  |  | (8.0%) | (8.6%) | (19.1%) | (17.1%) | (22.3%) | (2.6%) | (7.2%) | (22.4%) | (11.6%) | (41.1%) | 16.01 |
|  | E(C3) | 79 | 65 | 72 | 87 | 73 | 73 | 85 | 79 | 74 | 98 |  |
|  |  | (9.2%) | (7.1%) | (19.1%) | (14.5%) | (22.3%) | (3.9%) | (2.4%) | (17.9%) | (14.0%) | (34.2%) | 14.47 |
|  | E(C5) | 76 | 64 | 70 | 87 | 73 | 74 | 81 | 76 | 69 | 90 |  |
|  |  | (12.6%) | (8.6%) | (21.3%) | (14.5%) | (22.3%) | (2.6%) | (2.4%) | (13.4%) | (19.8%) | (23.3%) | 14.09 |
|  | E(L) | 84 | 65 | 74 | 89 | 73 | 74 | 95 | 86 | 82 | 112 |  |
|  |  | (3.4%) | (7.1%) | (16.9%) | (17.1%) | (22.3%) | (2.6%) | (14.5%) | (28.4%) | (4.7%) | (53.4%) | 17.04 |
|  | E(Q) | 85 | 56 | 68 | 107 | 75 | 82 | 122 | 105 | 89 | 144 |  |
|  |  | (2.3%) | (20.0%) | (23.6%) | (40.8%) | (20.2%) | (7.9%) | (47.0%) | (56.7%) | (3.5%) | (97.3%) | 31.92 |
| Other | O | 784 | 891 | 824 | 872 | 876 | 939 | 892 | 950 | 924 | 947 |  |
|  | E(C1) | 758 | 815 | 821 | 827 | 883 | 859 | 939 | 937 | 952 | 976 |  |
|  |  | (3.3%) | (8.5%) | (0.4%) | (5.2%) | (0.8%) | (8.5%) | (5.3%) | (1.4%) | (3.0%) | (3.1%) | 3.94 |
|  | E(C3) | 758 | 815 | 821 | 825 | 873 | 851 | 913 | 911 | 919 | 938 |  |
|  |  | (3.3%) | (8.5%) | (0.4%) | (5.4%) | (0.3%) | (9.4%) | (2.4%) | (4.1%) | (0.5%) | (1.0%) | 3.53 |
|  | E(C5) | 702 | 740 | 763 | 791 | 821 | 800 | 846 | 841 | 862 | 866 |  |
|  |  | (10.5%) | (16.9%) | (7.4%) | (9.3%) | (6.3%) | (14.8%) | (5.2%) | (11.5%) | (6.7%) | (8.6%) | 9.71 |
|  | E(L) | 812 | 888 | 877 | 860 | 936 | 911 | 1,015 | 1,016 | 1,020 | 1,063 |  |
|  |  | (3.6%) | (0.3%) | (6.4%) | (1.4%) | (6.8%) | (3.0%) | (13.8%) | (6.9%) | (10.4%) | (12.2%) | 6.49 |
|  | E(Q) | 732 | 777 | 796 | 826 | 930 | 900 | 1,119 | 1,117 | 1,204 | 1,259 |  |
|  |  | (6.6%) | (12.8%) | (3.4%) | (5.3%) | (6.2%) | (4.2%) | (25.4%) | (17.6%) | (30.3%) | (32.9%) | 14.47 |
