# Supplementary material for: Cancer incidence estimation from mortality data: a validation study within a population-based cancer registry
Source: Popul Health Metr. 2021 Mar 23;19:18. doi: 10.1186/s12963-021-00248-1 (PMC7988947; doi:10.1186/s12963-021-00248-1)
Supplement: Supplementary file 2 — Additional file 2: Table S2. Observed cases and expected cases under each scenario, and goodness of fit measures. Women. [file 12963_2021_248_MOESM2_ESM.docx]

**Table S2.** Observed cases and expected cases under each scenario, and goodness of fit measures. Women.

| Cancer site | Scenario | 2004 | 2005 | 2006 | 2007 | 2008 | 2009 | 2010 | 2011 | 2012 | 2013 | MAPE |
| --- | --- | --- | --- | --- | --- | --- | --- | --- | --- | --- | --- | --- |
| Colon | O | 117 | 138 | 135 | 153 | 144 | 153 | 152 | 144 | 168 | 163 |  |
|  | E(C1) | 132 | 145 | 142 | 117 | 118 | 143 | 165 | 174 | 187 | 184 |  |
|  |  | (12.8%) | (5.1%) | (5.2%) | (23.5%) | (18.1%) | (6.5%) | (8.6%) | (20.8%) | (11.3%) | (12.9%) | 12.48 |
|  | E(C3) | 131 | 145 | 142 | 118 | 118 | 138 | 158 | 168 | 186 | 186 |  |
|  |  | (12.0%) | (5.1%) | (5.2%) | (22.9%) | (18.1%) | (9.8%) | (3.9%) | (16.7%) | (10.7%) | (14.1%) | 11.84 |
|  | E(C5) | 123 | 131 | 130 | 118 | 113 | 132 | 150 | 163 | 175 | 178 |  |
|  |  | (5.1%) | (5.1%) | (3.7%) | (22.9%) | (21.5%) | (13.7%) | (1.3%) | (13.2%) | (4.2%) | (9.2%) | 9.99 |
|  | E(L) | 140 | 159 | 153 | 117 | 122 | 151 | 175 | 182 | 198 | 191 |  |
|  |  | (19.7%) | (15.2%) | (13.3%) | (23.5%) | (15.3%) | (1.3%) | (15.1%) | (26.4%) | (17.9%) | (17.2%) | 16.49 |
|  | E(Q) | 140 | 140 | 136 | 112 | 116 | 187 | 228 | 221 | 185 | 164 |  |
|  |  | (19.7%) | (1.4%) | (0.7%) | (26.8%) | (19.4%) | (22.2%) | (50.0%) | (53.5%) | (10.1%) | (0.6%) | 20.45 |
| Rectum | O | 67 | 61 | 75 | 60 | 67 | 70 | 70 | 77 | 66 | 56 |  |
|  | E(C1) | 53 | 42 | 31 | 50 | 81 | 66 | 65 | 86 | 73 | 96 |  |
|  |  | (20.9%) | (31.1%) | (58.7%) | (16.7%) | (20.9%) | (5.7%) | (7.1%) | (11.7%) | (10.6%) | (71.4%) | 25.49 |
|  | E(C3) | 58 | 47 | 35 | 50 | 73 | 63 | 60 | 78 | 67 | 84 |  |
|  |  | (13.4%) | (23.0%) | (53.3%) | (16.7%) | (9.0%) | (10.0%) | (14.3%) | (1.3%) | (1.5%) | (50.0%) | 19.24 |
|  | E(C5) | 45 | 39 | 30 | 42 | 60 | 52 | 61 | 82 | 62 | 75 |  |
|  |  | (32.8%) | (36.1%) | (60.0%) | (30.0%) | (10.4%) | (25.7%) | (12.9%) | (6.5%) | (6.1%) | (33.9%) | 25.44 |
|  | E(L) | 64 | 48 | 35 | 57 | 100 | 80 | 66 | 85 | 82 | 111 |  |
|  |  | (4.5%) | (21.3%) | (53.3%) | (5.0%) | (49.3%) | (14.3%) | (5.7%) | (10.4%) | (24.2%) | (98.2%) | 28.62 |
|  | E(Q) | 24 | 19 | 12 | 46 | 164 | 88 | 110 | 184 | 138 | 253 |  |
|  |  | (64.2%) | (68.9%) | (84.0%) | (23.3%) | (144.8%) | (25.7%) | (57.1%) | (139.0%) | (109.1%) | (351.8%) | 106.78 |
| Lung | O | 45 | 52 | 51 | 53 | 48 | 62 | 59 | 71 | 60 | 56 |  |
|  | E(C1) | 34 | 26 | 24 | 42 | 38 | 42 | 59 | 68 | 60 | 58 |  |
|  |  | (24.4%) | (50.0%) | (52.9%) | (20.8%) | (20.8%) | (32.3%) | (0.0%) | (4.2%) | (0.0%) | (3.6%) | 20.90 |
|  | E(C3) | 33 | 27 | 27 | 41 | 40 | 40 | 56 | 63 | 59 | 57 |  |
|  |  | (26.7%) | (48.1%) | (47.1%) | (22.6%) | (16.7%) | (35.5%) | (5.1%) | (11.3%) | (1.7%) | (1.8%) | 21.64 |
|  | E(C5) | 28 | 22 | 24 | 37 | 38 | 40 | 55 | 59 | 55 | 55 |  |
|  |  | (37.8%) | (57.7%) | (52.9%) | (30.2%) | (20.8%) | (35.5%) | (6.8%) | (16.9%) | (8.3%) | (1.8%) | 26.87 |
|  | E(L) | 40 | 31 | 26 | 46 | 40 | 44 | 61 | 74 | 64 | 61 |  |
|  |  | (11.1%) | (40.4%) | (49.0%) | (13.2%) | (16.7%) | (29.0%) | (3.4%) | (4.2%) | (6.7%) | (8.9%) | 18.26 |
|  | E(Q) | 34 | 15 | 10 | 41 | 28 | 58 | 90 | 111 | 70 | 62 |  |
|  |  | (24.4%) | (71.2%) | (80.4%) | (22.6%) | (41.7%) | (6.5%) | (52.5%) | (56.3%) | (16.7%) | (10.7%) | 38.30 |
| Breast | O | 338 | 345 | 389 | 410 | 415 | 494 | 419 | 466 | 450 | 494 |  |
|  | E(C1) | 314 | 405 | 469 | 543 | 480 | 466 | 428 | 354 | 342 | 374 |  |
|  |  | (7.1%) | (17.4%) | (20.6%) | (32.4%) | (15.7%) | (5.7%) | (2.1%) | (24.0%) | (24.0%) | (24.3%) | 17.33 |
|  | E(C3) | 294 | 375 | 437 | 505 | 472 | 465 | 437 | 369 | 351 | 377 |  |
|  |  | (13.0%) | (8.7%) | (12.3%) | (23.2%) | (13.7%) | (5.9%) | (4.3%) | (20.8%) | (22.0%) | (23.7%) | 14.76 |
|  | E(C5) | 235 | 283 | 324 | 365 | 355 | 364 | 352 | 305 | 301 | 325 |  |
|  |  | (30.5%) | (18.0%) | (16.7%) | (11.0%) | (14.5%) | (26.3%) | (16.0%) | (34.5%) | (33.1%) | (34.2%) | 23.48 |
|  | E(L) | 391 | 535 | 629 | 743 | 624 | 579 | 516 | 414 | 389 | 425 |  |
|  |  | (15.7%) | (55.1%) | (61.7%) | (81.2%) | (50.4%) | (17.2%) | (23.2%) | (11.2%) | (13.6%) | (14.0%) | 34.31 |
|  | E(Q) | 459 | 628 | 694 | 793 | 477 | 417 | 334 | 240 | 263 | 328 |  |
|  |  | (35.8%) | (82.0%) | (78.4%) | (93.4%) | (14.9%) | (15.6%) | (20.3%) | (48.5%) | (41.6%) | (33.6%) | 46.41 |
| Corpus uteri | O | 114 | 106 | 99 | 107 | 103 | 123 | 127 | 124 | 107 | 124 |  |
|  | E(C1) | 158 | 157 | 140 | 103 | 122 | 136 | 116 | 73 | 95 | 127 |  |
|  |  | (38.6%) | (48.1%) | (41.4%) | (3.7%) | (18.4%) | (10.6%) | (8.7%) | (41.1%) | (11.2%) | (2.4%) | 22.43 |
|  | E(C3) | 154 | 164 | 164 | 125 | 143 | 144 | 123 | 77 | 92 | 112 |  |
|  |  | (35.1%) | (54.7%) | (65.7%) | (16.8%) | (38.8%) | (17.1%) | (3.1%) | (37.9%) | (14.0%) | (9.7%) | 29.29 |
|  | E(C5) | 136 | 127 | 123 | 104 | 131 | 158 | 135 | 93 | 115 | 140 |  |
|  |  | (19.3%) | (19.8%) | (24.2%) | (2.8%) | (27.2%) | (28.5%) | (6.3%) | (25.0%) | (7.5%) | (12.9%) | 17.35 |
|  | E(L) | 178 | 193 | 171 | 114 | 125 | 122 | 106 | 61 | 78 | 109 |  |
|  |  | (56.1%) | (82.1%) | (72.7%) | (6.5%) | (21.4%) | (0.8%) | (16.5%) | (50.8%) | (27.1%) | (12.1%) | 34.62 |
|  | E(Q) | 175 | 100 | 36 | 23 | 36 | 90 | 78 | 54 | 135 | 357 |  |
|  |  | (53.5%) | (5.7%) | (63.6%) | (78.5%) | (65.0%) | (26.8%) | (38.6%) | (56.5%) | (26.2%) | (187.9%) | 60.23 |
| Ovary | O | 60 | 77 | 58 | 67 | 68 | 69 | 62 | 50 | 53 | 51 |  |
|  | E(C1) | 85 | 71 | 73 | 73 | 71 | 70 | 92 | 61 | 64 | 72 |  |
|  |  | (41.7%) | (7.8%) | (25.9%) | (9.0%) | (4.4%) | (1.4%) | (48.4%) | (22.0%) | (20.8%) | (41.2%) | 22.25 |
|  | E(C3) | 83 | 75 | 80 | 74 | 71 | 71 | 88 | 63 | 67 | 74 |  |
|  |  | (38.3%) | (2.6%) | (37.9%) | (10.4%) | (4.4%) | (2.9%) | (41.9%) | (26.0%) | (26.4%) | (45.1%) | 23.61 |
|  | E(C5) | 83 | 71 | 78 | 85 | 76 | 76 | 93 | 67 | 70 | 80 |  |
|  |  | (38.3%) | (7.8%) | (34.5%) | (26.9%) | (11.8%) | (10.1%) | (50.0%) | (34.0%) | (32.1%) | (56.9%) | 30.23 |
|  | E(L) | 86 | 74 | 73 | 64 | 67 | 65 | 89 | 57 | 61 | 67 |  |
|  |  | (43.3%) | (3.9%) | (25.9%) | (4.5%) | (1.5%) | (5.8%) | (43.5%) | (14.0%) | (15.1%) | (31.4%) | 18.89 |
|  | E(Q) | 103 | 46 | 37 | 65 | 78 | 68 | 141 | 46 | 46 | 64 |  |
|  |  | (71.7%) | (40.3%) | (36.2%) | (3.0%) | (14.7%) | (1.4%) | (127.4%) | (8.0%) | (13.2%) | (25.5%) | 34.14 |
| Other | O | 718 | 763 | 703 | 822 | 790 | 838 | 850 | 856 | 791 | 858 |  |
|  | E(C1) | 624 | 655 | 681 | 635 | 640 | 700 | 749 | 767 | 784 | 849 |  |
|  |  | (13.1%) | (14.2%) | (3.1%) | (22.7%) | (19.0%) | (16.5%) | (11.9%) | (10.4%) | (0.9%) | (1.0%) | 11.28 |
|  | E(C3) | 613 | 641 | 668 | 624 | 621 | 673 | 710 | 725 | 748 | 815 |  |
|  |  | (14.6%) | (16.0%) | (5.0%) | (24.1%) | (21.4%) | (19.7%) | (16.5%) | (15.3%) | (5.4%) | (5.0%) | 14.30 |
|  | E(C5) | 564 | 578 | 599 | 574 | 562 | 590 | 608 | 634 | 637 | 704 |  |
|  |  | (21.4%) | (24.2%) | (14.8%) | (30.2%) | (28.9%) | (29.6%) | (28.5%) | (25.9%) | (19.5%) | (17.9%) | 24.09 |
|  | E(L) | 677 | 725 | 756 | 688 | 707 | 798 | 877 | 882 | 919 | 983 |  |
|  |  | (5.7%) | (5.0%) | (7.5%) | (16.3%) | (10.5%) | (4.8%) | (3.2%) | (3.0%) | (16.2%) | (14.6%) | 8.68 |
|  | E(Q) | 684 | 731 | 744 | 697 | 770 | 876 | 1,033 | 1,092 | 1,036 | 1,078 |  |
|  |  | (4.7%) | (4.2%) | (5.8%) | (15.2%) | (2.5%) | (4.5%) | (21.5%) | (27.6%) | (31.0%) | (25.6%) | 14.27 |
