# Supplementary figures and images for: Cancer incidence estimation from mortality data: a validation study within a population-based cancer registry
Source: Popul Health Metr. 2021 Mar 23;19:18. doi: 10.1186/s12963-021-00248-1 (PMC7988947; doi:10.1186/s12963-021-00248-1)

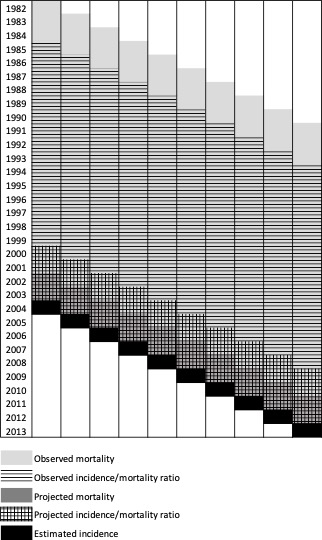

Supplement: Supplementary file 3 — Additional file 3: Fig. S1. Design of the data used in the iterative procedure to derived the incidence time series. [file 12963_2021_248_MOESM3_ESM.tiff]

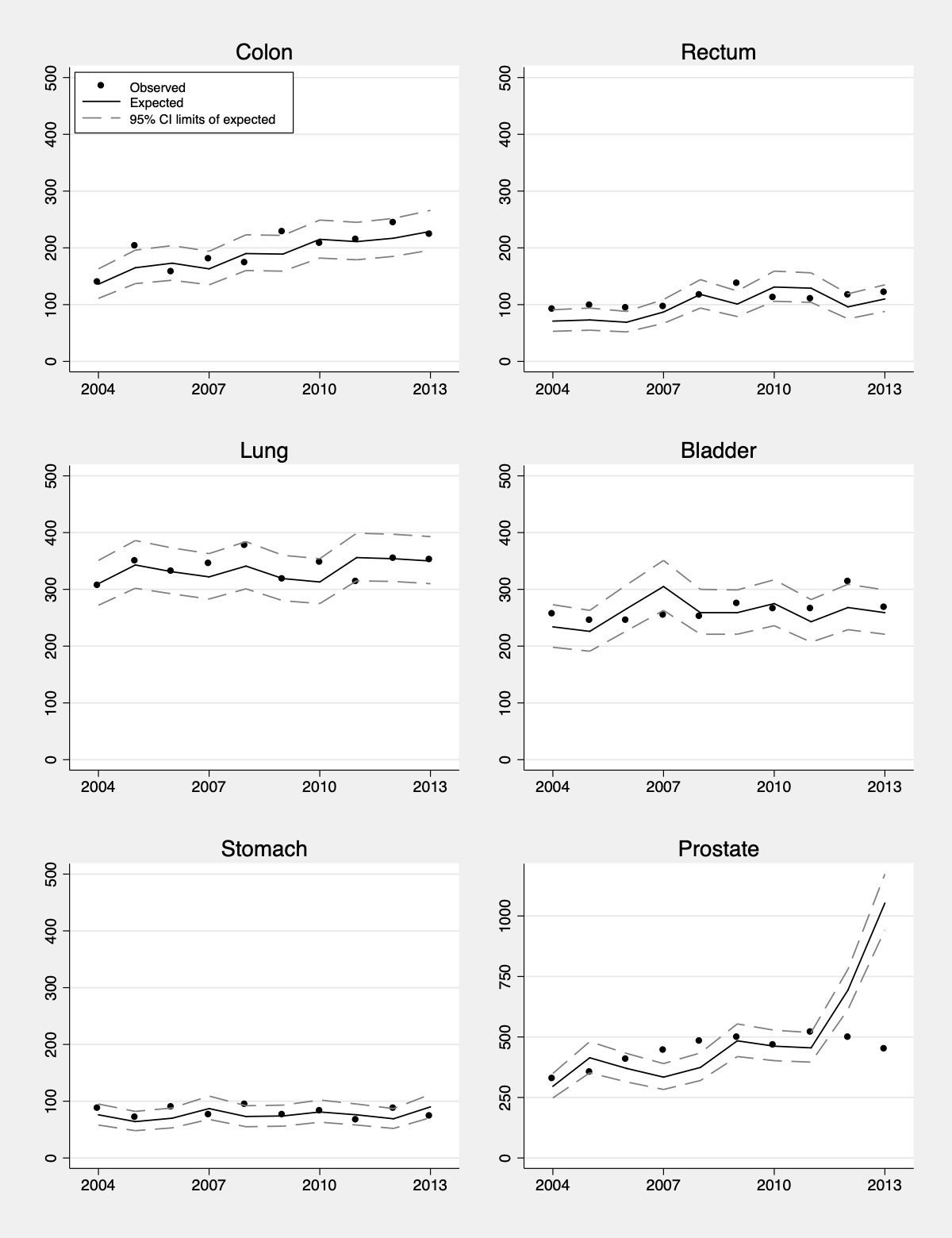

Supplement: Supplementary file 4 — Additional file 4: Fig. S2. Number of observed and expected (with 95% CI) cases under the best scenario for each site. Men. [file 12963_2021_248_MOESM4_ESM.png]

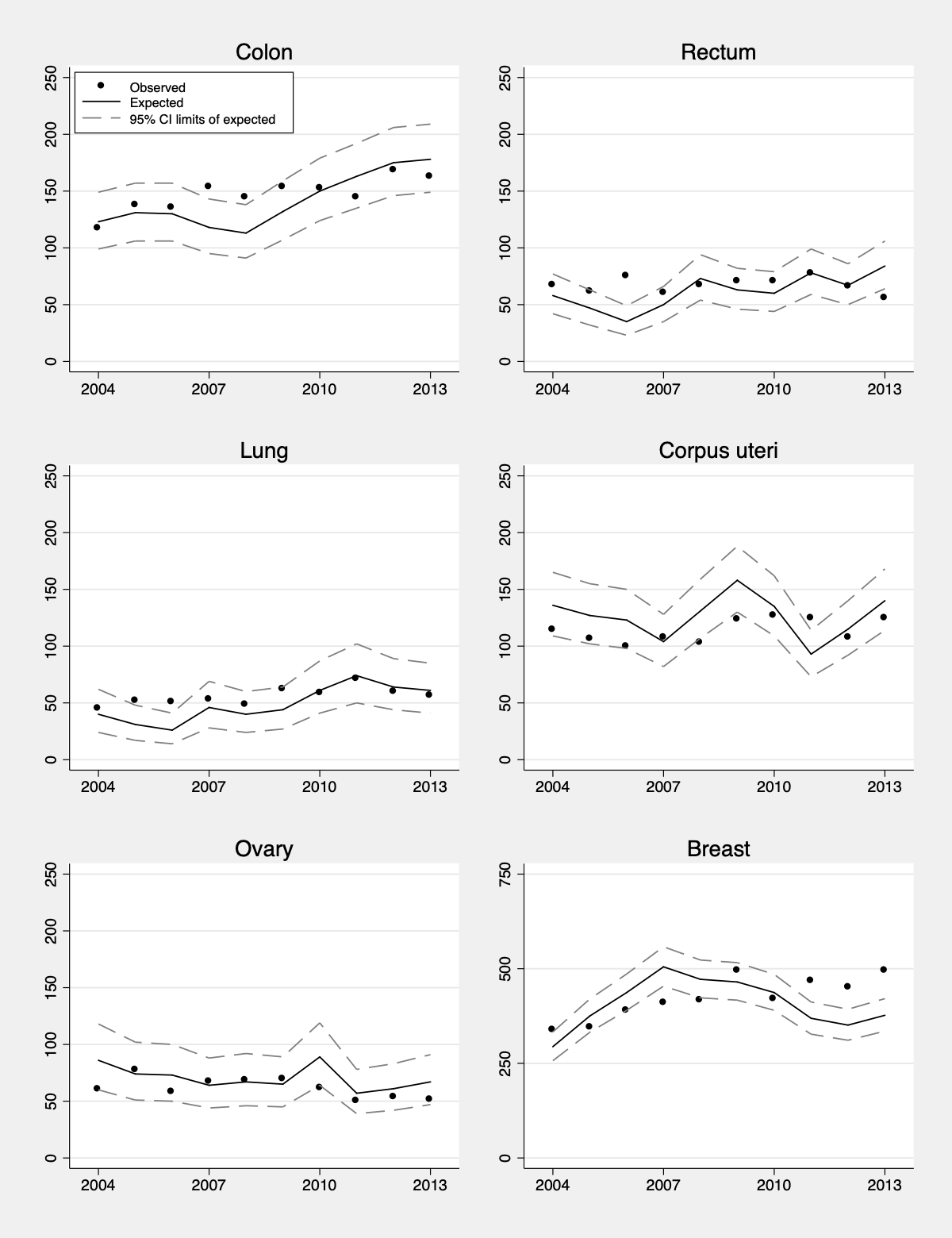

Supplement: Supplementary file 5 — Additional file 5: Fig. S3. Number of observed and expected (with 95% CI) cases under the best scenario for each site. Women. [file 12963_2021_248_MOESM5_ESM.png]
